# Fatigue in patients with Parkinson’s disease: a cross-sectional study

**DOI:** 10.3389/fnagi.2026.1837817

**Published:** 2026-07-15

**Authors:** Haibo Ning, Lei Chen, Min Wang, Ning Lv, Lihua Chen

**Affiliations:** 1 Nantong Haimen People's Hospital, Nantong, China; 2 Tianjin Medical University General Hospital, Tianjin, China; 3 Tianjin Huanhu Hospital, Tianjin, China; 4 Department of Neurology, Sinopharm North Hospital, Baotou, China

**Keywords:** associated factors, fatigue, motor symptoms, non-motor symptoms, Parkinson’s disease

## Abstract

**Objective:**

The purpose of this study is to ascertain the incidence of fatigue and to determine the cross-sectional associate and related variables of fatigue in patients with Parkinson’s disease (PD).

**Methods:**

A sample of 130 healthy individuals (39.23% male, mean age 64.19 ± 8.70 years) and 130 patients with PD (50.00% males, mean age 65.06 ± 9.04) were enrolled in the study. All participants were evaluated using the Fatigue Severity Scale (FSS), Mini-Mental State Examination (MMSE), Hamilton anxiety scale (HAMA), and Hamilton depression scale (HAMD). Patients with PD were assessed with the Unified Parkinson’s Disease Rating Scale (UPDRS), Hoehn & Yahr staging, the MMSE, HAMA, HAMD, the Parkinson’s Disease Sleep scale-2 (PDSS-2), and Parkinson’s Disease Questionnaire-39 (PDQ-39).

**Results:**

The PD group exhibited significantly higher scores in the FSS (*p* < 0.001) and demonstrated a greater susceptibility to fatigue compared to the control group. The FSS score in female patients was significantly higher than that in male patients (*p* = 0.003). Patients experiencing fatigue exhibited significantly higher scores on the UPDRS (*p* < 0.001), Hoehn & Yahr staging (*p* < *0*.001), NMSS (*p* < 0.001), HAMD (*p* < 0.001), HAMA (*p* < 0.001), PDSS-2 (*p* = 0.012), and PDQ-39 (*p* < 0.001) compared to those without fatigue. Correlation analysis revealed significant associations between fatigue and NMSS, HAMD, HAMA, PDSS, PDQ-39, UPDRS, and Hoehn & Yahr stage. In the FSS-based multiple linear regression, HAMA and PDQ-39 remained cross-sectional associated with higher fatigue severity, whereas NMSS showed a borderline effect. To directly evaluate multivariable discrimination for fatigue occurrence, we constructed a combined logistic model including NMSS, HAMD, and PDQ-39. The combined model showed good discrimination (AUC 0.879, 95% CI 0.806–0.941) and acceptable calibration (Brier score 0.125), outperforming any single scale.

**Conclusion:**

Patients with Parkinson’s disease experience a higher incidence of distressing fatigue compared to healthy individuals. The significant prevalence of fatigue among these patients is linked to various motor and non-motor symptoms.

## 1 Introduction

Parkinson’s disease (PD) is a chronic, progressive neurodegenerative disorder that primarily affects middle-aged and elderly individuals. Rigidity, tremor, and bradykinesia are the three main symptoms characterizing the motor impairments of PD. In addition to motor impairments, PD is also associated with various non-motor symptoms, including autonomic dysfunction, depression, chronic fatigue, and sleep disturbances. Among these manifestations, fatigue is a prevalent symptom among patients with PD. The prevalence of fatigue in PD is estimated to range from 33 to 70% (Nassif and Pereira, 2018). Approximately one in three patients with Parkinson’s disease regard fatigue as their most debilitating symptom (Friedman and Friedman, 1993), greatly restricting their daily functional capacity and independent living ability.

Fatigue is characterized by an overwhelming sense of tiredness, a lack of energy, and persistent exhaustion (Finsterer and Mahjoub, 2014). Patients experiencing pathological fatigue endure ongoing tiredness even when at rest, which severely impacts their daily lives and overall vitality. Jason *et al.* defined pathological fatigue as pathological lasting more than 3 months, with intensity surpassing previously experienced fatigue, compromising daily activities and quality of life (Jason et al., 2010). Fatigue may persist for over 6 months, classified as chronic fatigue, and can be associated with emotional or social disturbances (Kluger et al., 2016; Friedman et al., 2016), often accompanied by a pronounced sense of exhaustion. Fatigue is one of the non-motor symptoms of PD and has garnered increasing attention in recent years. It tends to manifest in the early stages of PD, including during the pre-motor phase. Several studies have indicated that fatigue is a leading cause of disability in PD, limiting patients’ ability to engage in hobbies and participate in social activities (Zesiewicz et al., 2007), and significantly reducing their quality of life (Dogan et al., 2015).

According to a meta-analysis, approximately half of patients with PD may experience fatigue (Siciliano et al., 2018). However, fatigue is often under-recognized because its clinical manifestations of fatigue can significantly overlap with those of depression and sleepiness, particularly in the advanced stages of PD. Various studies have indicated that fatigue is associated with multiple factors, including gender differences, disease duration, disease severity, disease stage, autonomic dysfunction, anxiety, depression, motor symptom severity, and levodopa equivalent dose. However, contradictory findings have been reported regarding the relationship between fatigue and other factors (Donzuso et al., 2023; Picillo et al., 2016; Havlikova et al., 2008; Pal et al., 2004; Hagell and Brundin, 2009; Beiske and Svensson, 2010; Cong et al., 2022; Skorvanek et al., 2015). Therefore, this research aimed to assess the factors influencing fatigue in PD patients and to investigate the correlation between fatigue and other non-motor symptoms. By identifying and analyzing these related factors, this study provides clinicians with insights for recognizing and managing fatigue in Parkinson’s disease, thereby contributing to an improved quality of life for patients.

## 2 Methods

### 2.1 Participants

130 patients (mean age 65.06 ± 9.04 years) who met the MDS diagnostic criteria for primary Parkinson’s disease (Postuma et al., 2015) were recruited in this study. The cohort included 65 males and 65 females, with ages ranging from 21 to 88 years. Patients with concomitant dementia (MMSE score < 23) or any other conditions potentially associated with fatigue, such as cardiac disease, malignancies, end-organ failure, severe orthopedic deformities affecting movement, or significant neurological or psychiatric disorders, were excluded from the study. Additionally, 130 healthy elderly controls without Parkinson’s disease (mean age 64.19 ± 8.70 years) were recruited from the same age range, comprising 51 males and 79 females. These controls were also screened to exclude any diseases that could be associated with fatigue. Statistical analysis indicated no significant differences in gender or age between the patients with PD and the healthy control group. All participants provided signed informed consent.

### 2.2 Assessment of fatigue

Patients were categorized into fatigue and non-fatigue groups based on the Fatigue Severity Scale (FSS). The FSS is the most widely utilized fatigue assessment tool in medical research and is the sole scale recommended by the MDS Working Group for screening and evaluating severity (Huether et al., 2023). The FSS comprises nine items, each rated on a scale from 1 (strongly disagree) to 7 (strongly agree), with patients asked to assess their level of fatigue using the same scale. The total FSS score is calculated as the average of the nine-item scores, ranging from 1 to 7. A cutoff value of ≥ 4 typically indicates significant fatigue (Siciliano et al., 2019), and the FSS endorsed for both screening and assessing the severity of fatigue.

### 2.3 Assessment of motor and non-motor symptoms

Motor symptoms were evaluated using the Unified Parkinson’s Disease Rating Scale (UPDRS) (Goetz et al., 2007). PD staging was conducted using the modified Hoehn and Yahr staging (Hoehn and Yahr, 1967). Non-motor symptoms were assessed using the following scales: the Non-Motor Symptoms Scale (NMSS) was used for screening non-motor symptoms, cognitive functioning was evaluated with the Montreal Cognitive Assessment (MoCA) and the Mini-Mental State Examination (MMSE), mood was measured using the 17-item Hamilton Depression Scale (HAMD) (Zheng et al., 1988; Kertzman et al., 2002), and anxiety was assessed with the 14-item Hamilton Anxiety Scale (HAMA) (Maier et al., 1988). Sleep disorders were evaluated using a modified version of the Parkinson’s Disease Sleep Scale-2 (PDSS-2), while quality of life was measured with the Parkinson’s Disease Questionnaire-39 (PDQ-39). Higher scores on all scales indicated greater severity of the assessed conditions.

This study aims to compare the clinical characteristics of cognitive function, mood, and fatigue under different measured conditions. We analyzed demographic data, including gender, age, disease duration, daily levodopa dosage equivalent intake, and UPDRS scores in the “ON” disease state of the disease, to assess the severity of motor symptoms in both fatigue and non-fatigue groups. Additionally, we compared non-motor symptoms, cognitive function, mood (measured by HAMA and HAMD), sleep quality, and quality of life between the two cohorts. This comparison focused on the severity of each parameter in both groups and examined the correlation between fatigue and other non-motor symptoms in PD.

### 2.4 Levodopa equivalent dose

The equivalent units of levodopa are calculated as follows: 100 mg levodopa = 2 mg cabergoline = 1 mg pergolide = 1 mg pramipexole = 5 mg ropinirole = 10 mg selegiline = 1 mg reserpine = 100 mg amantadine = 3.3 mg rotigotine = 300 mg entacapone. One tablet of Madopar = 200 mg levodopa, one tablet of Sinemet = 150 mg levodopa, 1 tablet of Stalevo = 141.6 mg levodopa (Schade et al., 2020). The following conversion factors were used for levodopa-controlled release and levodopa/carbidopa/entecapone combinations: 125 mg-controlled release levodopa = 65 mg levodopa;50 mg levodopa/carbidopa/entecapone = 65 mg levodopa.

## 3 Statistical methods

Statistical analysis was conducted using R4.0.3 software. The research design adopted a hierarchical correction strategy: the preset five core hypothesis tests (the differences in FSS, HAMD, HAMA, MMSE, and MOCA scores between the PD group and the control group)were corrected using the Holm-Bonferroni method (corrected significance threshold alpha = 0.01). Secondary analyses (gender/disease-duration subgroup comparison, correlation analysis, regression analysis, and predictive modeling) used the Benjamini-Hochberg method to control the false discovery rate (FDR, q = 0.1). Intergroup comparison of quantitative data was evaluated for normality by Shapiro–Wilk test and homogeneity of variance by Levene’s test. Independent sample t-test was used when data met parametric assumptions; otherwise, Wilcoxon rank sum test was used. Categorical data were analyzed using the chi-square test or Fisher’s exact test. Quantitative indicators are described as mean +/− standard deviation or median (Q1, Q3), and categorical indicators are described as counts and percentages. Spearman rank correlation analysis (FDR correction) was performed between the FSS score and NMSS, MMSE, MOCA, HAMD, HAMA, PDSS, PDQ-39, UPDRS-III, and H-Y stage. Multiple linear regression analysis was conducted with FSS score as the dependent variable and NMSS, HAMD, HAMA, PDQ-39, UPDRS-III, and H-Y stage as candidate predictors. Variance inflation factors (VIFs) were calculated to assess multicollinearity. To address reviewer concerns regarding predictive performance, a multivariable logistic model combining NMSS, HAMD, and PDQ-39 was additionally fitted for fatigue status (FSS > = 4), and its discrimination was summarized by ROC analysis while calibration was evaluated using a calibration plot and Brier score.

## 4 Results

### 4.1 Healthy control group *vs.* PD group

There was no statistically significant difference between the two groups in terms of gender (*p* = 0.105) and age (*p* = 0.430). However, a significant difference was observed in fatigue scores (*p* < 0.001) between the two groups (original *p* = 1.21 × 10^−5^, *p* = 0.002* after Holm correction). The median FSS score of the PD group was 2.00 (Q1 = 1.00, Q3 = 4.07), which was significantly higher than 1.22 (1.00, 2.00) of the control group. Additionally, the PD group exhibited significantly higher scores in depression (HAMD: original p = 3.31 × 10^−7^, adjusted p = 0.0005*), anxiety (HAMA: original p = 0.0005, adjusted p = 0.002*), and cognitive function (MMSE: The original p = 1.44 × 10^−5^ and the corrected p = 0.0003*) were both significantly inferior to the control group. These findings indicated that individuals with PD were more likely to have depressive and anxiety symptoms and cognitive impairments than those in the control group (Table 1).

**Table 1:** Comparison of clinical characteristics between control group and PD group.

Variable	Control group (*n* = 130)	Parkinson’s disease group (*n* = 130)	*p* value	The *p* value after Holm correction
Sex				
Male	51 (39.23%)	65 (50.00%)		
Female	79 (60.77%)	65 (50.00%)	0.105^a^	
Age (yr)	64.19 (8.70)	65.06 (9.04)	0.430^b^	
FSS	1.22 (1.00,2.00)	2.00(1.00,4.07)	**<0.001** ^**c**^	0.002*
HAMD	4.31 (4.74)	7.33 (4.78)	**<0.001** ^**b**^	0.0005*
HAMA	6.90 (5.30)	9.20 (5.86)	**<0.001** ^**b**^	0.002*
MMSE	28.18 (2.17)	26.98 (2.39)	**<0.001** ^**b**^	0.0003*

Variables are expressed as means (standard deviations), except for gender number (percentages), FSS [median (interquartile range)].

All differences are calculated with the ^b^independent-sample t tests except for ^a^Chi square test and ^c^Mann–Whitney U test.

FSS = Fatigue Severity Scale; HAMD = Hamilton depression scale; HAMA = Hamilton anxiety scale; MMSE = Mini-Mental State Examination.

Bold values indicate significant difference. *p* < 0.05. *q < 0.05.

### 4.2 Female *vs.* male subgroup comparisons with PD

Among 130 patients with PD, 38 cases were accompanied by fatigue (FSS ≥ 4), accounting for 29.23%. Among them, 26 were female and 12 were male, with a male–female ratio of 1:2.17. There were significant differences in gender (original *p* = 0.0122) and age (original *p* = 0.0139) between the two groups of PD with fatigue (PDF) and without fatigue (PDNF), but there was no statistically significant difference in the disease course (original *p* = 0.276). Since female patients were more prone to fatigue symptoms (68.42% of females in the PDF group), we grouped and analyzed 130 PD patients by gender. The results showed that: The female group was significantly inferior to the male group in the scores of MOCA (original *p* = 0.0103, adjusted q = 0.030*), HAMA (original *p* = 0.0029, adjusted q = 0.009*) and PDQ-39 (original *p* = 0.0017, adjusted q = 0.005*), indicating that female patients exhibited more severe depressive symptoms and poorer overall quality of life (Table 2).

**Table 2:** Comparison of clinical characteristics between female and male patients with PD.

Variable	Male (*n* = 65)	Female (*n* = 65)	*p* value	q value after FDR correction
Age (yr)	64.78 (8.01)	65.54 (10.05)	0.630^a^	
Disease duration (yr)	3.27 (2.00)	3.19 (2.00)	0.814^a^	
FSS	1.42 (1.00, 3.05)	2.89 (1.28, 4.47)	**0.003** ^**b**^	
UPDRS I	3.02 (3.03)	3.26 (2.47)	0.604^a^	
UPDRS II	10.38 (6.18)	10.54 (6.31)	0.874^a^	
UPDRS III	22.93 (15.64)	25.64 (15.33)	0.113^a^	
H&Y	2.00(1.00, 2.00)	2.00 (1.00, 2.50)	0.373^b^	
NMSS	31.30 (22.39)	35.21 (25.98)	0.359^a^	
MMSE	27.00 (25.75, 29.00)	27.00 (22.75, 29.00)	0.374^b^	
MOCA	22.86 (3.94)	20.69 (5.54)	**0.010** ^**a**^	0.030^*^
HAMA	7.63 (5.54)	10.68 (5.98)	**0.003** ^**a**^	0.009^*^
HAMD	6.75 (4.74)	7.85 (5.01)	0.197^a^	
PDSS-2	19.00 (7.50, 18.00)	28.00 (10.50, 48.00)	0.303^b^	
PDQ-39	18.66 (18.25)	30.16 (22.87)	**0.002** ^**a**^	0.005^*^

Variables are expressed as means (standard deviations), except for gender number (percentages), H&Y, MMSE and PDSS-2 [median (interquartile range)].

All differences are calculated with the ^a^independent-sample t tests except for ^b^Mann–Whitney U test.

FSS = Fatigue Severity Scale; UPDRS = Unified Parkinson’s Disease Rating Scale; H&Y stage = Hoehn & Yahr staging; NMSS = non-motor symptoms scale; MMSE = Mini-Mental State Examination; MOCA = Montreal Cognitive Assessment; HAMA = Hamilton anxiety scale; HAMD = Hamilton depression scale; PDSS-2 = Parkinson’s Disease Sleep scale-2; PDQ-39 = Parkinson’s Disease Questionnaire-39.

Bold values indicate significant difference. *p* < 0.05. *q < 0.05.

### 4.3 Fatigue *vs.* non-fatigue subgroup comparisons with PD

Subgroup analysis of PD with fatigue (PDF) and without fatigue (PDNF): In the PDF group, UPDRSIII (original *p* = 0.0004241, corrected q = 0.001*), H-Y stage (original *p* = 0.0002986, corrected q = 0.001*), and NMSS (original *p* = 2.03 × 10^−7^). After correction, q = 0.000001* and other indicators were significantly inferior to those of the PDNF group (Table 3). There was no significant difference in MMSE, MOCA and LED between the two groups (*p* > 0.05) (Table 3).

**Table 3:** Clinical characteristics of patients with fatigue and without fatigue.

Variable	With fatigue (*n* = 38)	Without fatigue (*n* = 92)	*p* value	q value after FDR correction
Sex				0.036*
Male	12 (31.58%)	53 (57.61%)		
Female	26 (68.42%)	39 (42.39%)	**0.012** ^**b**^	
Age (yr)	67.76 (7.10)	63.95 (9.54)	**0.014** ^**a**^	0.020*
Disease duration (yr)	3.63 (2.65)	3.15 (2.03)	0.276^a^	
UPDRS I	4.47 (3.23)	3.054 (3.73)	**0.016** ^**a**^	
UPDRS II	13.76 (6.18)	8.87 (5.30)	**<0.001** ^**a**^	
UPDRS III	33.97 (18.02)	22.33 (14.91)	**<0.001** ^**a**^	0.001*
H&Y	2.25 (1.50,3.00)	2.00 (1.00, 2.00)	**<0.001** ^**c**^	0.000448*
NMSS	49.550 (24.131)	24.700 (17.085)	**<0.001** ^**a**^	0.000001*
MMSE	28.00 (26.00, 29.00)	27.00 (25.00, 29.00)	0.335^c^	
MOCA	21.89 (3.64)	22.38 (4.56)	0.524^a^	
HAMA	13.18 (5.54)	7.56 (5.18)	**<0.001** ^**a**^	
HAMD	10.47 (4.80)	6.03 (4.15)	**<0.001** ^**a**^	
PDSS-2	32.50 (18.00, 46.00)	24.04 (6.00, 48.25)	**0.012** ^**c**^	
PDQ-39	39.71 (21.24)	17.62 (16.91)	**<0.001** ^**a**^	
LDE	472.90 (216.49)	408.50 (201.787)	0.120^a^	

Variables are expressed as means (standard deviations), except for gender number (percentages), H&Y, MMSE and PDSS-2 [median (interquartile range)].

All differences are calculated with the ^a^independent-sample t tests except for ^b^Chi square test and ^c^Mann–Whitney U test.

UPDRS = Unified Parkinson’s Disease Rating Scale; H&Y stage = Hoehn & Yahr staging; NMSS = non-motor symptoms scale; MMSE = Mini-Mental State Examination; MOCA = Montreal Cognitive Assessment; HAMA = Hamilton anxiety scale; HAMD = Hamilton depression scale; PDSS-2 = Parkinson’s Disease Sleep scale-2; PDQ-39 = Parkinson’s Disease Questionnaire-39; LDE = Levodopa dosage equivalent.

Bold values indicate significant difference. *p* < 0.05. *q < 0.05.

### 4.4 Factors associated with fatigue

**Figure 1:**
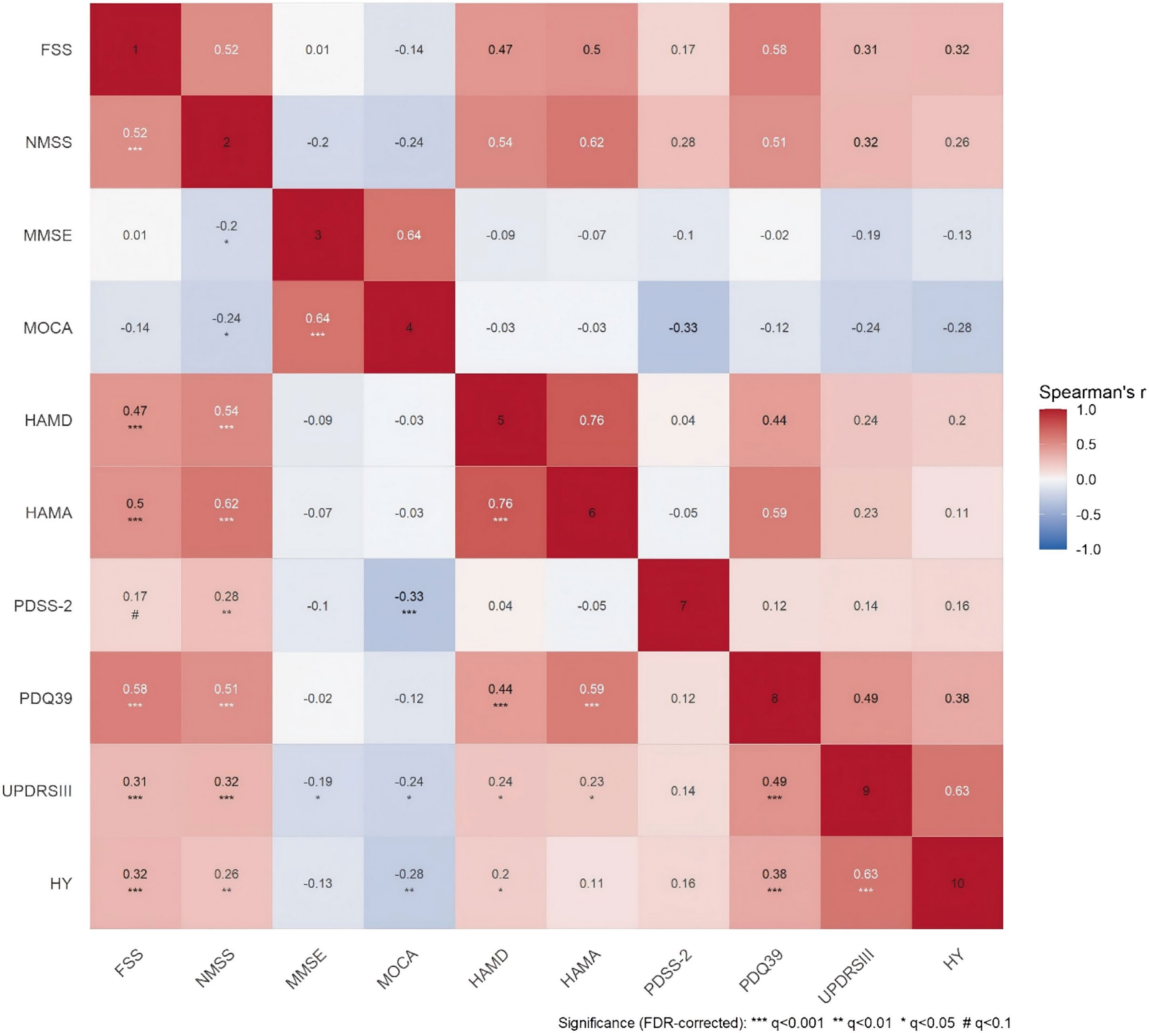
Heatmap of Spearman correlation coefficients among clinical variables in patients with Parkinson’s disease. Color intensity indicates the absolute value of correlation coefficients (red: positive correlation; blue: negative correlation). Values within each cell represent Spearman’s *ρ*. Statistical significance was corrected by the false discovery rate (FDR): *** q < 0.001, ** q < 0.01, * q < 0.05, # q < 0.1. FSS, Fatigue Severity Scale; NMSS, Non-Motor Symptoms Scale; MMSE, Mini-Mental State Examination; MoCA, Montreal Cognitive Assessment; HAMD, Hamilton Depression Rating Scale; HAMA, Hamilton Anxiety Rating Scale; PDSS-2, Parkinson’s Disease Sleep Scale-2; PDQ-39, 39-Item Parkinson’s Disease Questionnaire; UPDRS-III, Unified Parkinson’s Disease Rating Scale Part III; H&Y, Hoehn & Yahr stage.

Correlation analysis revealed that higher scores on the NMSS, HAMD, HAMA, PDSS-2, PDQ-39, UPDRS Part III, and Hoehn & Yahr stage were significantly associated with an increased risk of clinically distressing fatigue in patients with Parkinson’s disease. These clinical indicators were strongly correlated with fatigue severity and FSS total scores (Table 4). Figure 1 presents the Spearman correlation heatmap of all enrolled clinical variables. Color intensity is proportional to the absolute value of the correlation coefficient, with red indicating positive correlations and blue indicating negative correlations; a blue–white–red divergent color scale was adopted for visualization.

**Table 4:** Analysis of the correlation between FSS scores and other factors.

Variable	Spearman ρ	Raw P value	FDR q value	Significance
PDQ-39	0.582	<0.001	<0.001	***
NMSS	0.516	<0.001	<0.001	***
HAMA	0.497	<0.001	<0.001	***
HAMD	0.466	<0.001	<0.001	***
H&Y	0.317	<0.001	<0.001	***
UPDRS-III	0.307	<0.001	<0.001	***
PDSS-2	0.170	0.054	0.069	#
MOCA	−0.142	0.107	0.121	ns
MMSE	0.008	0.928	0.928	ns

***q < 0.001, **q < 0.01, *q < 0.05, # q < 0.1 (Significant edge), ns: No statistical significance.

### 4.5 Factors associated with fatigue

To further explore the determinants of fatigue severity measured by the Fatigue Severity Scale (FSS) in patients with Parkinson’s disease (PD), multiple linear regression analysis was performed. The FSS score was set as the dependent variable, while NMSS, HAMD, HAMA, PDQ-39, UPDRS III, and Hoehn-Yahr stage were entered as candidate predictors (Table 5).

**Table 5:** Multiple linear regression analysis of related factors with FSS scores.

Variable	Beta value	*p* value	95% CI
NMSS	0.012	0.060	−0.001–0.025
HAMD	0.010	0.795	−0.065–0.084
HAMA	0.071	0.031	0.006–0.135
PDQ-39	0.021	0.008	0.005–0.036
UPDRS III	−0.004	0.678	−0.022–0.014
H&Y	0.205	0.225	−0.126-0.536

UPDRS = Unified Parkinson’s Disease Rating Scale; H&Y stage = Hoehn & Yahr staging; NMSS = non-motor symptoms scale; HAMA = Hamilton anxiety scale; HAMD = Hamilton depression scale; PDQ-39 = Parkinson’s Disease Questionnaire-39.

Multicollinearity was formally assessed using variance inflation factors (VIFs). All predictors showed low-to-moderate VIF values (range 1.84–3.03), indicating no concerning multicollinearity and no need for further variable reduction (Table 6). In the FSS-based multiple linear regression, HAMA (beta = 0.071, *p* = 0.031) and PDQ-39 (beta = 0.021, *p* = 0.008) remained independently associated with greater fatigue severity, whereas NMSS showed a borderline effect (beta = 0.012, *p* = 0.060). The adjusted R2 of the model was 0.410.

**Table 6:** Multicollinearity diagnosis (VIF values) for predictors of fatigue severity (FSS score).

Variable	VIF value	Interpretation
NMSS	2.03	VIF < 5
HAMD	2.51	VIF < 5
HAMA	3.16	VIF < 5
PDQ-39	2.35	VIF < 5
UPDRS-III	1.95	VIF < 5
H&Y	1.92	VIF < 5

**Figure 2:**
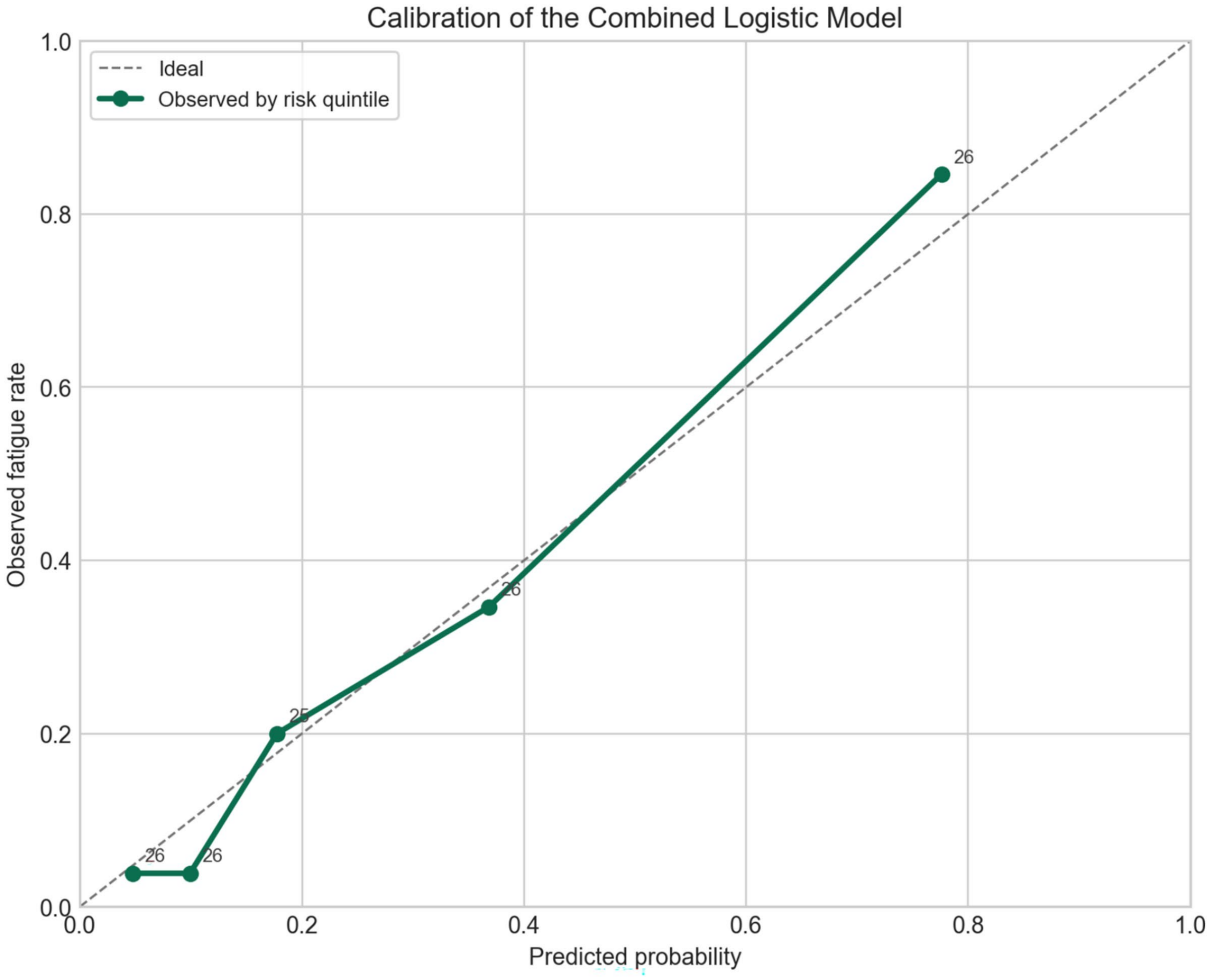
Calibration plot of the combined logistic model for fatigue in PD. PD = Parkinson’s disease; NMSS = Non-Motor Symptoms Scale; HAMD = Hamilton depression scale; PDQ-39 = Parkinson’s Disease Questionnaire-39.

To directly evaluate multivariable discrimination for fatigue occurrence, we additionally fitted a combined logistic model including NMSS, HAMD, and PDQ-39 among complete cases. In this model, all three variables were independently associated with fatigue status (Table 7). The combined model achieved an AUC of 0.879 (95% CI 0.806–0.941), with sensitivity 0.737 and specificity 0.912 at the optimal Youden threshold, outperforming the single-scale ROC analyses (Table 8; Figure 2). The calibration plot showed acceptable agreement between predicted and observed risk strata, with a Brier score of 0.125 (Figure 2).

**Table 7:** Multivariable logistic regression model for fatigue status in PD.

Variable	Beta value	OR	95% CI for OR
NMSS	0.028	1.029	1.004–1.054
HAMD	0.139	1.149	1.008–1.309
PDQ-39	0.037	1.037	1.010–1.065
AUC/Brier	—	—	AUC 0.879 (0.806–0.941); Brier 0.125
Trio VIF	NMSS 1.84	HAMD 1.56	All < 5

**Table 8:** ROC performance of the individual scales and the combined multivariable model.

Model	AUC (95% CI)	Sensitivity	Specificity	Cut-off	SE	Youden’s index	*p* value
Combo	0.879 (0.806–0.941)	0.737	0.912	0.399	0.038	0.649	<0.001
NMSS	0.833 (0.752–0.905)	0.921	0.626	26.000	0.044	0.547	<0.001
PDQ-39	0.836 (0.759–0.903)	0.789	0.802	25.000	0.043	0.592	<0.001
HAMD	**0.787** (0.691–0.873)	0.737	0.780	9.000	0.048	0.517	<0.001
HAMA	**0.800** (0.711–0.883)	0.737	0.747	11.000	0.047	0.484	<0.001
MMSE	**0.498** (0.392–0.608)	0.132	0.923	21.000	0.056	0.055	0.967

AUC = area under the curve; 95% CI = 95% confidence interval; SE = standard error; NMSS = Non-Motor Symptoms Scale; HAMD = Hamilton depression scale; HAMA = Hamilton anxiety scale; MMSE = Mini-Mental State Examination; PDQ-39 = Parkinson’s Disease Questionnaire-39. Youden’s index was used to determine the optimal threshold for each model. Bold values indicate the best discriminative performance.

### 4.6 Predictive performance of the combined multivariable model

**Figure 3:**
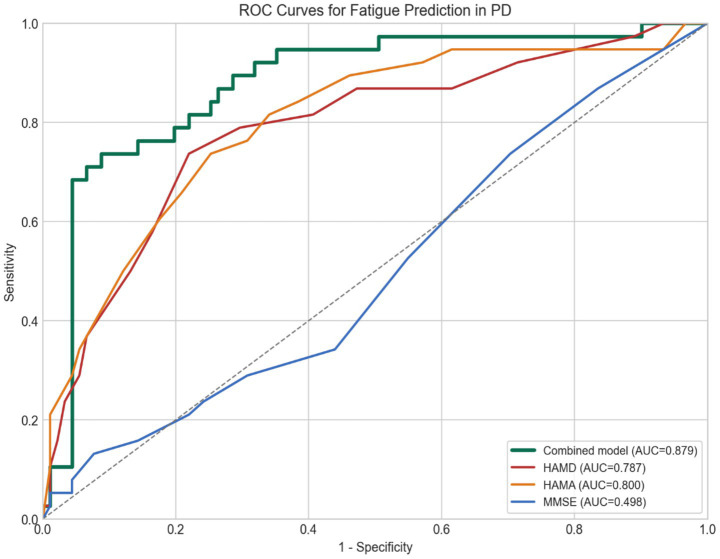
Receiver operating characteristic (ROC) curves for the combined model and single scales.

ROC analysis was performed to compare the discriminative performance of the combined multivariable model with individual scales. The combined model that integrated NMSS, HAMD, and PDQ-39 showed the highest discrimination (AUC 0.879), exceeding that of HAMD (AUC 0.787), HAMA (AUC 0.800), and MMSE (AUC 0.498). Table 8 summarizes the ROC characteristics of the individual and combined models, and Figure 3 shows the corresponding ROC curves.

## 5 Discussion

Our research aimed to evaluate the relationship between fatigue and multiple factors, including gender, disease severity, disease stage, motor symptom severity, and non-motor symptoms such as anxiety, depression, cognitive disturbances, sleep disturbances, and quality of life. We sought to confirm the correlation between fatigue and these non-motor symptoms. In this study, among 130 patients with Parkinson’s disease, 29.23% were accompanied by fatigue, suggesting that fatigue is a common non-motor symptom in PD. The fatigue frequency of PD patients in this study was consistent with that of previous studies (Beiske and Svensson, 2010; Cong et al., 2022). In our study, when comparing and analyzing the demographic data of the two groups, both gender and age were statistically significant, and elderly patients had more cases of fatigue. Among patients with Parkinson’s disease, the degree of fatigue in female patients is significantly higher than that in male patients, which is to some extent consistent with a previous study in Norway (Skorvanek et al., 2015). Patients with PD in the fatigue group were older, exhibited a greater degree of motor impairment and disease severity, reported poorer sleep quality and overall quality of life, and were more likely to have mood disorders such as depression and anxiety, as well as increased cognitive impairment. Our report found that there was a strong correlation between the FSS score and several other indicators, including the NMSS score, HAMD score, HAMA score, PDSS score, PDQ-39 score, UPDRS-III score and H-Y stage. However, further multiple linear regression analysis of the related factors revealed that the UPDRS-III score and H&Y stage could not predict the occurrence of fatigue in PD patients. This indirectly suggests that there is no direct relationship between the motor symptoms and fatigue manifestations of PD patients. Some studies have confirmed that fatigue can occur at all stages of PD, even before the appearance of motor symptoms (Ou et al., 2021). In the FSS-based multiple linear regression, PDQ-39 and HAMA remained independently associated with greater fatigue severity, while NMSS showed a borderline contribution. Importantly, formal multicollinearity diagnostics did not show concerning VIF values, suggesting that these symptom domains could be examined jointly without unstable coefficient inflation. To further explore their correlative effects, we established a combined multivariate model incorporating NMSS, HAMD and PDQ-39. This integrated model exhibited superior discriminatory ability compared with individual scales and had good fitting consistency. The findings suggested that combining indicators of non-motor symptoms, depressive symptoms and quality of life can better reflect fatigue status in PD patients than using a single scale alone.

PD fatigue and gender: There is growing evidence that PD affects women and men differently, with documented differences in epidemiology, clinical features, and treatment responses (Meoni et al., 2020). A higher incidence of PD and a more severe progression have been reported among men. However, as the disease progresses, women are at higher risk of developing highly disabling motor complications, such as motor fluctuations and dyskinesias (Picillo et al., 2017). In our study, we found that the FSS score in female patients was significantly higher than that in male patients (Table 2), and female patients were more likely to present with fatigue symptoms than male patients and with a greater PD with fatigue (PDF) subgroup (Table 3), which is consistent to some extent with a previous Norwegian study (Beiske and Svensson, 2010). There was no significant difference between the two groups comparing the levodopa equivalence daily dose, suggesting that fatigue is not related to medication (Havlikova et al., 2008; Herlofson et al., 2012; Valko et al., 2010). It was found that men’s trigger for fatigue was more likely to be physical exertion, while for women, they may be physiological factors, emotional factors, and differences in social roles (Lin et al., 2021). From a physiological perspective, for example, the regulatory effects of estrogen and progesterone on the central nervous system (such as the 5-HT energy system), And the influence of specific physiological stages of women such as the menstrual cycle, pregnancy, and menopause on fatigue perception; In undertaking social roles, women often undertake more unpaid labor (such as housework and parenting), and this “second occupation” is often overlooked, which may lead to the accumulation of chronic fatigue, On the other hand, women have a higher need for emotional regulation, and women with Parkinson’s disease are more likely to experience depressive symptoms. Consistent with prior evidence, female patients tend to present with more severe depressive symptoms and worse overall quality of life, potentially explaining the higher fatigue burden observed in female individuals (Zhu et al., 2016). Meanwhile, disease duration did not differ significantly between the two groups. This may be due to the limited sample size, and further studies with larger samples are warranted.

PD fatigue and depression: It is important to note that depression is significantly correlated with fatigue, indicating that as the severity of depression increases, so do the accompanying fatigue symptoms. This correlation is likely because both depression and anxiety heighten the subjective perception of fatigue in patients. Several epidemiological studies have reported an association between depression and fatigue in patients with PD, although some individuals may experience fatigue without accompanying depression (Berardelli et al., 2012). In a 10-year follow-up study, patients experiencing fatigue exhibited significantly higher scores for depressive symptoms (Berardelli et al., 2012). In our study, multiple linear regression analysis revealed a significant correlation between depression and fatigue. Numerous studies have demonstrated that serotonergic dysfunction in patients with PD is linked to the development of both fatigue and depression (Chaudhuri and Behan, 2000; Politis and Niccolini, 2015), suggesting that these symptoms may share a common neurobiological mechanism (Politis and Niccolini, 2015). Research has also indicated that depression may accelerate the onset of fatigue (Hagell and Brundin, 2009; Friedman et al., 2011), and can exacerbate subjective perceptions of fatigue (Friedman et al., 2007). Pavese et al. confirmed that the severity of fatigue was significantly negatively correlated with cerebrospinal fluid levels of serotonin (5-HT) (Pavese et al., 2010). Using multiple logistic regression, the presence of fatigue was predicted only by depressive symptoms, depression is not the main cause of high-frequency fatigue in PD patients in a 10-year follow-up study, 43.5% of patients without depression problems still reported fatigue, patients with fatigue had significantly higher scores of depressive symptoms. In this sample, the prevalence of fatigue in patients without depressive symptoms or sleepiness increased from 32.1% in 1993 to 38.9% in 2001. This study shows that fatigue can be separated from depression (and sleepiness) and that fatigue is highly prevalent in the nondepressed sample (Beiske and Svensson, 2010). The complex interaction and overlap of fatigue with other PD symptoms may reflect the lack of pathophysiological knowledge of fatigue, but it also reflects the low specificity in evaluating this unclear symptom.

In the present study, the combined model including NMSS, HAMD, and PDQ-39 achieved an AUC of 0.879, which was clearly higher than the AUCs of HAMD (0.787), HAMA (0.800), and MMSE (0.498) alone. This result directly supports the view that fatigue in PD is multidimensional and cannot be adequately summarized by a single affective or cognitive scale. The calibration plot also suggested acceptable agreement between predicted and observed fatigue risk strata, with a Brier score of 0.125. From a clinical perspective, these findings imply that a combined assessment of non-motor burden, depressive symptoms, and quality of life may be more useful for fatigue screening than any single questionnaire.

PD fatigue and Cognition: The cognitive changes in Parkinson’s disease are obvious in almost all individuals, including visuospatial, memory retrieval, positional shift and attention deficit, all of which can occur in the early stage of the disease. Besides selective cognitive impairment, 40 to 80% of PD patients will eventually develop Parkinson’s disease dementia It has not been determined yet whether the cognitive needs of PD patients lead to fatigue or whether fatigue leads to cognitive deficits. Due to the use of self-reported scales, most studies excluded PD subjects with dementia. Three studies observed the relationship between fatigue and cognitive function in PD patients and at least showed a cross-sectional relationship between fatigue and cognition. A small study showed that there was an association among FSS scores, depression and reduced frontal lobe perfusion (Abe et al., 2000). Rochester and his colleagues demonstrated (Okuma et al., 2009). That in walking activities that require more attention, physical fatigue is associated with a slower gait, suggesting that mental challenges may lead to fatigue and pain rather than being the result of physical exhaustion fatigue. A recent study in Norway reported the longitudinal fatigue process of PD patients during an 8-year follow-up period and found a relationship between a reduction in mini-mental state examinations and an increase in fatigue complaints (Kertzman et al., 2002)_._

The MMSE was adopted as the inclusion criterion to enroll PD patients without overt cognitive dysfunction. Nonetheless, many participants deemed cognitively normal by the MMSE had MoCA scores below the standard MCI cutoff, showing obvious inconsistency between the two cognitive scales. This discrepancy may be partly due to elderly patients’ limited tolerance to neuropsychological testing. The MoCA involves more cognitively demanding visuospatial, comprehension and reasoning items, which tend to lower test performance in older adults.

Merely attributing subthreshold MoCA scores to poor test tolerance is an oversimplified empirical interpretation rather than a rigorous inference. Two limitations should be noted. First, borderline low MoCA scores may reflect undetected subclinical MCI, acting as an unmeasured confounder that interferes with the correlation analysis between fatigue and cognitive function. Second, minor deviations in standardized MoCA administration may also result in unreliable borderline scores.

Although age-related test tolerance and scale characteristics can plausibly explain the MMSE–MoCA discrepancy, the potential effects of subclinical cognitive impairment and subtle measurement bias cannot be fully excluded. Future studies should strengthen cognitive phenotyping, standardize MoCA administration, and apply multi-timepoint assessments to reduce confounding and improve the reliability of exploring the fatigue–cognition relationship in PD patients.

PD fatigue and levodopa drug therapy: The relationship between fatigue and drug treatment: Levodopa preparations can improve the clinical symptoms of Parkinson’s patients, despite the increasing number of new drugs for treating Parkinson’s. But so far, no specific drug has been proposed to be effective for Parkinson’s fatigue syndrome. Levodopa, especially dopamine agonists, may cause insomnia or fragmented sleep (such as frequent dreams and increased awakenings at night), leading to daytime fatigue. The relationship between levodopa and PD fatigue is bidirectional. It may relieve fatigue by improving motor function, or it may aggravate fatigue due to fluctuations in drug efficacy or side effects. The first and most important step in dealing with PD patients with fatigue is to explain to them and their family that this is an important and common symptom of PD. They must also be made aware that this is a symptom that unfortunately cannot be treated effectively.

## 6 Conclusion

Our research has several limitations. The respondents of the study were mainly Chinese, with a small sample size and a lack of longitudinal follow-up data. The results may not be widely applicable to all ethnic groups. Detailed dosages of individual antiparkinsonian medications were not collected in this study, restricting further analysis of dose-dependent associations between dopaminergic drugs and fatigue. Nevertheless, we calculated the unified levodopa equivalent dose (LDE) for all participants, and no significant intergroup difference in LDE was found, as shown in Section 1.4 and Table 3. In addition, this study did not differentiate multiple dimensions of fatigue (physical, mental and emotional), nor did it survey specific exercise types used by patients to cope with fatigue. Moreover, the purpose of this study excluded fatigue in PD patients with dementia or other related severe clinical conditions. Finally, due to the observational and cross-sectional nature of this study, we are unable to draw conclusions regarding the associated causal relationship.

In summary, fatigue is one of the most prevalent non-motor symptoms experienced by patients with Parkinson’s disease. Female patients are more likely to report symptoms of fatigue than their male counterparts. Patients with PD who experience fatigue tend to be older and exhibit more severe motor impairments and disease progression, along with a greater number of non-motor symptoms, including poorer sleep quality and overall quality of life. Furthermore, patients in the fatigue group are more likely to suffer from disorders such as depression and anxiety, demonstrating greater impairment. A multiple regression analysis of factors influencing fatigue revealed that FSS was associated with non-motor symptom factors, including the severity of NMSS, the Non-Motor Symptoms Scale (NMSS), depression, and lower quality of life. Therefore, it is of great interest to explore how managing common non-motor symptoms can alleviate fatigue in patients with PD.

## Data Availability

The original contributions presented in the study are included in the article/supplementary material, further inquiries can be directed to the corresponding author.
